# Why Clinical Trials of Microbiome-Targeted Interventions Often Fail to Support Health Claims: A Commentary on Probiotics and Translational Design

**DOI:** 10.3390/microorganisms14020470

**Published:** 2026-02-14

**Authors:** Raul de Jesus Cano, Gissel García Menéndez

**Affiliations:** 1 Chauvell, LLC, San Luis Obispo, CA 93405, USA; 2 Pathology Department, Hospital Clínico y Quirúrgico Hermanos Ameijeiras, La Habana 10400, Cuba; gisselgarcia2805@gmail.com

**Keywords:** microbiome, probiotics, nutraceuticals, clinical trial design, translational research, biomarker strategy

## Abstract

The rapid expansion of probiotics and other microbiome-modulating interventions has been accompanied by a growing number of human clinical trials. However, despite frequent reports of statistically significant microbiome changes, relatively few studies generate evidence that convincingly supports health claims or translates into reproducible, clinically meaningful outcomes. This gap is often attributed to the inherent complexity and inter-individual variability of the gut microbiome; however, recurring shortcomings in trial design and interpretation likely play an equally important role. In this Commentary, we examine common failure modes that weaken the clinical validation of microbiome-mediated interventions. These include overreliance on descriptive microbiome metrics (e.g., alpha diversity and taxonomic shifts) as surrogate endpoints, misalignment between prespecified endpoints and the claims ultimately advanced, and excessive dependence on symptom-only outcomes in settings characterized by substantial placebo responsiveness. We further highlight how inadequate control of key confounders—particularly diet, antibiotic exposure, and concomitant medications—combined with endpoint overload and underpowered study designs, can obscure true biological signal and increase the risk of irreproducible findings. We argue that stronger evidence emerges when the microbiome is treated as a mechanistic mediator rather than a clinical endpoint. Trials are most interpretable when intended claims are prospectively defined, linked to explicit biological mechanisms, and evaluated using a hierarchy of endpoints that prioritizes host-relevant outcomes and objective biomarkers, with microbiome measures integrated to support mechanistic plausibility. Adoption of staged development pathways disciplined statistical planning, and transparent management of confounding variables can further improve reproducibility and clinical relevance.

## 1. Introduction

Over the past decade, the human gut microbiome has become a central focus of probiotic, nutraceutical, and functional food research. Advances in high-throughput sequencing, metagenomics, and bioinformatics have enabled increasingly detailed characterization of microbial communities and their responses to dietary and microbial interventions. In parallel, commercial and clinical interest in probiotics and other microbiome-modulating products has expanded rapidly, driven by their proposed therapeutic and preventive potential across a wide range of conditions, including gastrointestinal disorders, metabolic disease, immune dysfunction, and systemic [1,2,3].

This growth has been accompanied by a substantial increase in human clinical trials investigating probiotics, prebiotics, synbiotics, and related interventions. Many of these studies report statistically significant changes in gut microbiome composition, often interpreted as evidence of clinical benefit or improved gut health [4,5,6]. However, despite this growing body of literature, relatively few microbiome-focused trials generate evidence that convincingly supports health claims, withstands regulatory scrutiny, or translates into reproducible clinical outcomes. Discrepancies between microbiome modulation and clinically meaningful benefit remain common, contributing to uncertainty among clinicians, regulators, and scientifically literate consumers regarding the true therapeutic value of many probiotic interventions [7,8,9].

These limitations are frequently attributed to the inherent complexity and inter-individual variability of the gut microbiome. While biological heterogeneity is undeniably a challenge, it does not fully explain the recurring difficulty in validating probiotic and microbiome-targeted interventions. Increasingly, attention has turned to the design, execution, and interpretation of clinical trials themselves. Across diverse indications and intervention types, common methodological patterns emerge that limit interpretability and weaken the evidentiary basis for clinical claims, even when trials are well intentioned and technically competent [10,11,12].

A particularly pervasive issue is the reliance on descriptive microbiome metrics—such as alpha diversity, relative taxonomic abundance, or shifts in selected genera—as primary or surrogate endpoints for health benefit. Although these measures provide valuable insight into microbial community structure, they do not inherently reflect microbial function, host–microbe interactions, or physiological relevance. As a result, statistically significant microbiome changes may occur without corresponding improvements in clinical outcomes, leading to ambiguity in interpretation and overextension of claims [13,14,15]. Similar challenges arise when trial endpoints are poorly aligned with intended claims, when subjective symptom measures are not corroborated by objective biological markers, or when key confounding variables such as diet, antibiotic exposure, and baseline health status are insufficiently controlled.

At the same time, expectations for clinical validation are rising. Regulatory agencies, journal reviewers, and healthcare professionals increasingly demand biologically plausible mechanisms linking probiotic interventions to host benefit, along with transparent, reproducible evidence supporting claimed effects [16,17,18]. In this context, trials that prioritize convenience, exploratory outcomes, or post hoc interpretation over prospectively defined, mechanism-driven hypotheses face growing skepticism, regardless of statistical significance.

In this Commentary, we argue that many of the shortcomings observed in probiotic and microbiome-focused clinical trials are not inevitable consequences of biological complexity but rather reflect modifiable choices in study design and interpretation. Drawing on patterns observed across the published literature and practical experience in clinical research, we examine several recurring design and analytical pitfalls that limit the translational relevance of microbiome studies. We further discuss how greater alignment between intended claims, biological mechanisms, endpoint selection, and statistical discipline can improve the quality and interpretability of clinical validation efforts.

By focusing on principles rather than specific products or indications, this Commentary aims to provide a practical framework for strengthening the design of probiotic and microbiome-targeted clinical trials. As the field continues to mature, greater methodological rigor will be essential to realizing the therapeutic and preventive potential of probiotics and to ensuring that clinical applications are supported by evidence that is both scientifically credible and clinically meaningful.

## 2. Descriptive Microbiome Metrics Versus Functional Relevance

A pervasive limitation in microbiome-focused probiotic and nutraceutical trials is the reliance on descriptive microbial metrics—such as alpha diversity, taxonomic shifts, or the enrichment of “beneficial” taxa—as surrogate indicators of therapeutic success. Comparable limitations are evident in the vaginal microbiota (VMB), where traditional models emphasize Lactobacillus dominance as a universal marker of vaginal health, despite evidence that the VMB is highly dynamic across puberty, reproductive years, and menopause and that clinically relevant dysbiosis may occur even when Lactobacilli remain abundant. These observations underscore that taxonomic composition alone is insufficient for defining functional or clinical status and highlight the need for mechanism-aligned, host-relevant endpoints when evaluating microbiome-targeted interventions.

While these endpoints yield insight into community structure, their relevance to host physiology is frequently inferred rather than empirically demonstrated. This disconnect compromises the translational strength of many otherwise well-executed studies. Alpha diversity, for example, is often framed as a proxy for microbiome health or resilience, yet its interpretation is context-dependent and may not reflect meaningful changes in digestion, metabolism, immune modulation, or clinical symptoms. In certain settings—such as inflammatory or dysbiotic states—greater diversity may even indicate microbial instability or maladaptation. Similarly, changes in taxonomic composition may fail to reflect functional consequences due to strain-level variability, gene redundancy, and context-specific gene expression [13,14,19]. Moreover, a taxon’s presence alone does not guarantee functional relevance; microbial competence during host colonization depends on the organism’s metabolic capabilities and ecological fitness in the host environment. For example, the ability to produce short-chain fatty acids, compete for mucosal substrates, or modulate immune signaling pathways often varies at the strain level and is shaped by both host and community context.

In addition to context dependence, alpha diversity and relative-abundance metrics are constrained by the compositional nature of sequencing data. Unlike transcriptomics or proteomics, which provide approximate measures of absolute concentration, 16S and shotgun metagenomic sequencing generate relative-abundance profiles that are inherently interdependent and subject to compositional bias. As a result, apparent changes in one taxon’s relative abundance may reflect shifts in others rather than true biological expansion. Technical artifacts—such as variation in DNA-extraction efficiency, sequencing depth, and batch effects—can further introduce noise, leading to spurious correlations or misinterpretation of biological significance. Several high-profile trials illustrate this issue: in colorectal cancer patients, Hibberd et al. reported increased diversity and Lactobacillus abundance after probiotic intervention but did not measure host immune or tumor-related outcomes, precluding causal inference, and Ishikawa et al. observed similar microbial shifts with prebiotic fiber supplementation but likewise lacked direct host-level mechanistic endpoints [20,21].

Several high-profile trials illustrate this issue. In colorectal cancer patients, Hibberd et al. [22] reported increased diversity and *Lactobacillus* abundance after probiotic intervention but did not measure host-immune- or tumor-related outcomes, precluding causal inference. Ishikawa et al. [23] observed similar microbial shifts with prebiotic fiber supplementation but did not assess inflammation or metabolic markers. Other studies emphasized diversity preservation during antibiotic treatment [24] or taxonomic recovery following dietary or probiotic interventions [25] yet still omitted direct clinical endpoints such as infection rates, metabolic markers, or quality-of-life measures.

A central challenge in translational microbiome research lies in the overreliance on descriptive microbial endpoints—such as alpha diversity, relative taxonomic shifts, or the abundance of presumed “beneficial” taxa—as surrogates for clinical benefit. While these metrics offer insight into community structure, their relevance to health outcomes is often inferred rather than empirically demonstrated. This disconnect undermines the translational value of many probiotic and nutraceutical trials and limits their ability to support credible clinical claims.

For example, in a randomized trial of a multistrain probiotic in colorectal cancer patients [22], increased microbial diversity and enrichment of Lactobacillus species were interpreted as beneficial changes. However, no clinical endpoints related to tumor progression or immune function were evaluated, and no mechanistic pathways were tested to link microbiome alterations to cancer-related outcomes. Similarly, a study by Ishikawa et al. [26] reported increased diversity and elevated Bifidobacterium abundance in patients consuming prebiotic fiber but failed to assess host biomarkers of inflammation or metabolism, thereby limiting causal interpretation.

Descriptive measures are also context dependent. In some cases, higher diversity may not indicate improved health and could reflect microbial instability or dysbiosis. Moreover, taxonomic changes may not reflect functional impact due to gene redundancy, strain-level variability, and context-specific expression profiles [27]. This underscores the risk of interpreting taxonomic abundance shifts as functional or clinical effects in the absence of corroborating host-level data.

A summary of representative trials exemplifying these pitfalls is provided in Table 1. These cases illustrate how descriptive endpoints, while informative for exploratory insight, often fall short of supporting therapeutic claims when not paired with mechanistic or host-relevant outcomes.

The core problem is not that descriptive metrics lack value—they are indispensable for hypothesis generation, mechanistic exploration, and contextualizing findings. Rather, the problem arises when they are elevated to primary outcome status or interpreted as therapeutic proxies in the absence of supporting host-level evidence. Such trials risk being statistically rigorous but biologically ambiguous.

To enhance translational relevance, future trials should anchor microbiome measures within a framework that includes:

• Functional microbial endpoints (e.g., short-chain fatty acid production, bile acid transformation, metatranscriptomics).
• Relevant host biomarkers (e.g., CRP, insulin sensitivity, intestinal permeability).
• Clearly defined clinical outcomes aligned with the intended health claim.

Integrating functional and host-centric measures strengthens biological plausibility, supports causal inference, and enhances regulatory and clinical interpretability. This approach also ensures that microbiome shifts are not interpreted as health benefits per se but as mechanistic intermediaries through which interventions exert their effects—a critical distinction for advancing microbiome-based therapeutic development.

## 3. Misalignment Between Trial Endpoints and Intended Claims

A common limitation in probiotic and microbiome-focused trials is the mismatch between selected endpoints and the health claims they aim to support. In many cases, claims are formulated post hoc—after data collection—based on which outcomes reach statistical significance. While convenient, this retrospective strategy weakens the evidentiary foundation for clinical validation.

This issue arises across application areas. Trials focused on gut microbiome composition are often interpreted as supporting systemic or immune-related benefits, despite lacking direct measures of metabolic, inflammatory, or clinical endpoints. Inferring broad claims from stool-based microbiome data—without accompanying host-level evidence—limits interpretability and credibility.

For example, in a randomized controlled trial of fermented foods, Wastyk et al. [30] reported a 20% increase in gut microbial diversity (Shannon index) alongside a 19-marker reduction in systemic inflammatory cytokines in the intervention group—but without mechanistic linkage between these endpoints [30]. Similarly, John et al. [24] found that a probiotic preserved microbiota alpha diversity during antibiotic treatment (no significant post-treatment drop vs. a 25% decline in placebo), but they did not measure antibiotic-associated diarrhea or related host outcomes [24]. In a multistrain probiotic study for metabolic syndrome, Wastyk et al. [31] reported that while 30% of participants showed reduced triglycerides and blood pressure, these improvements were observed only in a post hoc defined “responder” subgroup, with no overall treatment effect at the cohort level [31].

Robust clinical validation requires endpoints that are both statistically sound and biologically relevant. Metrics selected for convenience or exploratory interest may yield publishable data, but they often fail to substantiate therapeutic or preventive effects. This challenge is especially pronounced in probiotic research, where mechanisms are multifactorial and highly context dependent.

Prospective alignment between claims, mechanisms, and endpoints is essential. Investigators should clearly define the intended benefit, articulate a plausible biological pathway, and select endpoints that capture both mechanistic engagement and clinical relevance. This hierarchy reduces reliance on post hoc narratives and supports stronger causal inference [38,39,40].

Lack of alignment also complicates statistical interpretation. Without clear endpoint prioritization, studies may be underpowered for critical outcomes while inflating the risk of false positives. Regulatory bodies and journals increasingly expect pre-specified designs that directly test the stated claim [41,42].

This does not preclude exploratory endpoints, which are valuable in early-stage research. However, they must be clearly labeled and interpreted with appropriate caution. When exploratory data are promoted to primary evidence, conclusions may exceed what the data can reasonably support. Distinguishing between hypothesis-generating and claim-validating outcomes is critical for maintaining scientific and clinical integrity.

Trials designed with disciplined claim definition, biologically grounded endpoints, and integrated mechanistic context are far better positioned to generate evidence that is interpretable, reproducible, and suitable for clinical or regulatory validation.

## 4. Symptom-Based Outcomes, Placebo Effects, and Responsible Interpretation

Patient-reported outcomes (PROs) are central to many probiotic and microbiome-focused trials, particularly in domains such as digestive health, functional gastrointestinal disorders, and subjective well-being. Since symptom relief is often the primary reason individuals seek probiotics, PROs can reflect meaningful clinical benefits. However, when used in isolation, symptom-based outcomes present interpretive challenges—especially in the context of strong placebo responsiveness.

Placebo-related symptom improvement is well-documented in nutraceutical and microbiome trials. These responses may stem from expectancy effects, regression to the mean, increased symptom awareness, or behavioral changes associated with trial participation [43,44,45]. In probiotic studies, placebo responsiveness may be especially pronounced, as participants frequently enroll with strong beliefs regarding gut health and anticipated benefit [5]. As a result, symptom improvement in both active and placebo arms should be viewed as an expected design reality rather than an anomaly.

Importantly, placebo-associated improvement does not invalidate a trial or negate the potential biological activity of an intervention. Rather, it underscores the limitations of using symptom change alone to establish clinical relevance. When symptom-based outcomes improve across study arms, differentiation between specific intervention effects and nonspecific responses becomes difficult in the absence of objective biological corroboration. This challenge is compounded by the inherent variability of symptom scales, differences in questionnaire sensitivity, and limited reproducibility across studies and populations [46,47].

From both scientific and regulatory perspectives, symptom-only outcomes are rarely sufficient to support clinical claims. Without anchoring to host-level biomarkers or mechanistic evidence, it remains unclear whether improvements reflect physiological modulation, transient perceptual shifts, or contextual artifacts unrelated to the intervention.

To address this, robust trials integrate PROs with objective host biomarkers that reflect relevant biological processes—such as inflammation, gut permeability, metabolic function, or immune activation. When symptom relief coincides with plausible changes in host biology—and, where available, functionally consistent microbiome shifts—confidence in causality is strengthened [48,49].

Such integration also enhances interpretation of placebo effects. Parallel symptom improvement across groups may highlight limitations in endpoint specificity, whereas divergence in biological markers can help isolate specific effects of the intervention. Rather than viewing placebo responsiveness as a nuisance, trials that incorporate biomarker context can use it as an interpretive tool to clarify which outcomes likely reflect true physiological change [50].

In summary, addressing placebo effects does not require excluding subjective outcomes, but contextualizing them appropriately. Trials that combine symptom-based endpoints with mechanism-aligned, objective markers are better positioned to generate evidence that is interpretable, reproducible, and clinically meaningful.

## 5. Interacting Pitfalls in Microbiome Trial Design

The common pitfalls in microbiome-focused trials—such as reliance on surrogate endpoints, statistical underpowering, and inadequate control for confounders—rarely occur in isolation. Instead, they interact in compounding ways, creating a self-reinforcing cycle that undermines both interpretability and translational relevance. For instance, small sample sizes may limit the ability to adjust for covariates like baseline microbiome composition or medication use, introducing uncontrolled confounding. If the study also relies on exploratory or poorly aligned endpoints, even statistically significant changes may lack clinical relevance or mechanistic coherence. These limitations often result in ambiguous findings that cannot support therapeutic claims or regulatory validation.

**Figure 1:**
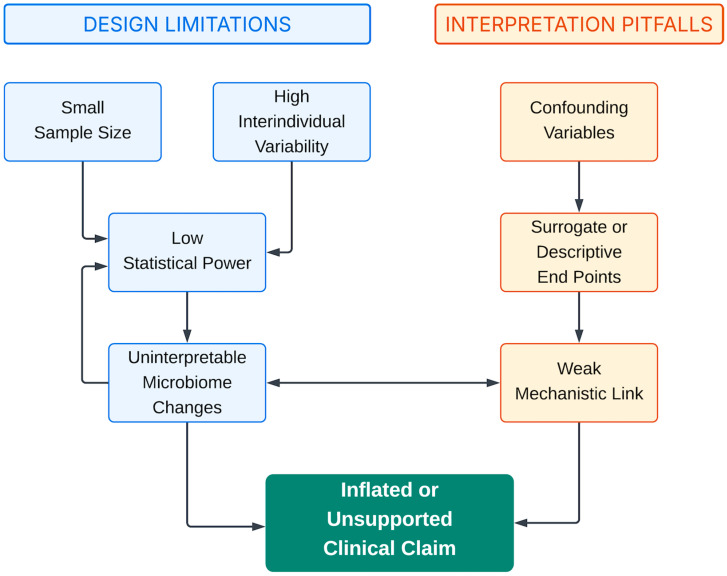
Compounding Pitfalls in Microbiome Trial Design. Common methodological flaws—such as low statistical power, reliance on surrogate or exploratory endpoints, inadequate adjustment for confounders, and microbiome findings of uncertain significance—frequently co-occur and reinforce one another. Their interaction creates a self-perpetuating cycle that limits interpretability, weakens causal inference, and undermines the translational relevance of study findings.

Figure 1 illustrates this dynamic interplay, highlighting how design flaws such as low power, confounding, endpoint misalignment, and microbiome ambiguity feed into one another. Recognizing these systemic interactions is essential to avoid circular logic and enhance the methodological rigor of microbiome intervention research.

## 6. Confounding Variables and Trial Discipline in Microbiome Research

A fundamental challenge in probiotic and microbiome-focused clinical trials is the sensitivity of the gut microbiome to a wide range of external and host-related variables. Diet, recent antibiotic exposure, concomitant medications, baseline health status, and lifestyle factors can all exert substantial influence on microbial composition and function. When these variables are insufficiently controlled or characterized, they introduce noise that can obscure true intervention effects and undermine interpretability.

Dietary intake represents one of the most significant confounders in microbiome research. Short-term changes in macronutrient composition, fiber intake, and food diversity can rapidly alter microbial community structure and metabolic output, sometimes to a greater extent than the intervention under study [51,52,53]. Dietary intake represents one of the most significant confounders in microbiome research. Short-term changes in macronutrient composition, fiber intake, and food diversity can rapidly alter microbial community structure and metabolic output, sometimes to a greater extent than the intervention under study. Trials that do not monitor or standardize diet risk attributing microbiome changes to the probiotic intervention when they may instead reflect dietary variability. While full dietary control is often impractical, failure to assess or account for dietary patterns limits the strength of causal inference.

Recent and concurrent antibiotic exposure poses an additional challenge. Antibiotics can induce profound and sometimes prolonged disruptions of the gut microbiome, affecting both taxonomic composition and functional capacity [54,55]. Inclusion of participants with heterogeneous antibiotic histories without appropriate stratification or exclusion criteria can introduce substantial inter-individual variability, reducing statistical power and complicating interpretation. Similar concerns apply to commonly used medications such as proton pump inhibitors, metformin, and nonsteroidal anti-inflammatory drugs, all of which have been shown to influence the gut microbiome independently of probiotic supplementation [56,57,58].

Baseline health status and microbiome composition further contribute to heterogeneity. Illustrative clinical data reinforce this point: a recent analysis of over 300 fecal samples found that individuals with type 2 diabetes frequently present with an altered Firmicutes/Bacteroidetes ratio—often below the conventional dysbiosis threshold of 0.8—accompanied by a disproportionate increase in Proteobacteria (median ~6.7%, IQR 3.6–17.3%). Dysbiosis type was significantly associated with T2D status (χ^2^ *p* = 0.033; OR ≈ 1.86), and sex-stratified analyses revealed that T2D females with dysbiosis had a markedly higher prevalence of cystitis/candidiasis (*p* < 0.01; OR ≈ 3.6). These findings underscore how metabolic status and sex-specific physiology shape baseline microbiome composition and contribute to clinical heterogeneity at trial entry.

Individuals may differ markedly in microbial diversity, functional potential, immune tone, and metabolic state at study entry, influencing both responsiveness to probiotic interventions and the direction of observed effects. Oxidative stress, shaped by lifestyle factors such as diet quality, physical activity, stress exposure, and sleep hygiene, represents an additional variable with the potential to influence both microbiome composition and host response. Its significance may be amplified by participant age, sex, and underlying health conditions. Furthermore, the duration of the trial must be sufficient to capture both microbiome remodeling and downstream host responses, as short intervention windows may underestimate or mischaracterize true biological effects in the context of this complex, multivariate background [59,60].

Beyond participant characteristics, trial discipline itself plays a critical role in data quality. Inadequate protocol adherence, inconsistent sample collection, variable timing of assessments, and incomplete follow-up can all erode signal and reduce confidence in study outcomes. Microbiome analyses are particularly sensitive to such inconsistencies, as sample handling, storage conditions, and sequencing workflows can introduce technical variability that compounds biological noise [61,62,63].

Addressing these challenges requires treating potential confounders as design parameters rather than statistical afterthoughts. This includes the use of clear inclusion and exclusion criteria, pre-specified handling of recent antibiotic use, dietary assessment or standardization strategies, and rigorous protocol adherence monitoring. While no trial can eliminate all sources of variability, transparent documentation and proactive management of confounding factors substantially enhance interpretability and reproducibility.

For probiotic interventions intended to support clinical applications, failure to adequately control or account for confounding variables represents a major barrier to validation. Trials that incorporate disciplined design practices—combined with thoughtful baseline characterization and high-quality execution—are more likely to detect true biological effects and generate evidence that is credible, reproducible, and clinically meaningful.

## 7. Sequencing Methodology as a Critical Variable in Microbiome Trials

The validity and interpretability of microbiome findings in clinical trials depend not only on biological variability but also on methodological rigor in sequencing strategy. Different sequencing approaches—such as 16S rRNA gene sequencing, shotgun metagenomics, and metatranscriptomics—offer varying levels of taxonomic and functional resolution. For example, 16S sequencing typically limits identification to the genus level and lacks functional insights, while shotgun metagenomics enables strain-level resolution and metabolic pathway reconstruction but requires greater depth, cost, and computational complexity [64].

Beyond platform choice, technical variables such as DNA extraction methods, primer selection, library preparation, and sequencing depth can introduce substantial variability across studies. Batch effects, sequencing artifacts, and differences in bioinformatic pipelines (e.g., OTU clustering versus ASV resolution, choice of reference databases) can further affect reproducibility and comparability [21,63]. The compositional nature of sequencing data introduces analytical challenges that require appropriate normalization and interpretation frameworks to avoid spurious associations.

To strengthen evidence claims, trials should report methodological details transparently, use validated protocols where available, and consider cross-platform validation when functional interpretation is critical. Where possible, integration of functional readouts—such as microbial metabolite profiling, metagenomics-informed pathway analysis, or strain-level dynamics—can bridge the gap between community shifts and host outcomes.

## 8. Statistical Power, Microbiome Variability, and Hypothesis Discipline

High inter-individual variability is a defining characteristic of the human gut microbiome and presents a substantial statistical challenge for probiotic and nutraceutical clinical trials. Microbial community composition, functional capacity, and host–microbe interactions vary widely across individuals, even within ostensibly homogeneous populations. When this variability is not adequately accounted for in study design and analysis, trials may be underpowered to detect biologically meaningful effects or may generate results that are difficult to reproduce.

Many microbiome-focused trials enroll sample sizes that are sufficient for detecting large effect sizes in conventional clinical biomarkers but inadequate for capturing more subtle or heterogeneous microbiome-mediated effects. This limitation is often compounded by the inclusion of numerous exploratory endpoints without clear prioritization. As the number of measured outcomes increases, statistical power for any single endpoint decreases, while the risk of false-positive findings rises. In such settings, statistically significant results may reflect chance associations rather than robust intervention effects [41,63,65].

Endpoint overload also complicates interpretation. Trials that attempt to measure a broad array of microbiome, biomarker, and symptom outcomes without a clearly defined primary hypothesis can produce complex datasets that are difficult to analyze coherently. Without pre-specified endpoint hierarchies and statistical plans, investigators may be tempted to emphasize outcomes that reach nominal significance while downplaying null findings. This practice, while often unintentional, undermines confidence in reported effects and contributes to inconsistency across studies.

Hypothesis discipline is therefore critical. Well-designed trials begin with a limited number of clearly articulated primary hypotheses tied directly to the intended claim and underlying biological mechanism. Sample size calculations should be based on realistic effect size assumptions for these primary outcomes, informed by prior data where available. Secondary and exploratory endpoints can provide valuable context and support mechanistic interpretation, but they should be explicitly designated as such and interpreted cautiously [66,67].

Pilot-to-pivotal development pathways offer a pragmatic strategy for addressing uncertainty in effect size and variability. Early-stage pilot studies can be used to assess feasibility, refine endpoints, and generate preliminary estimates of variability, which in turn inform the design of larger, confirmatory trials. Attempting to combine exploratory discovery and definitive validation within a single, underpowered study often leads to ambiguous results that satisfy neither objective.

From a clinical validation perspective, statistical rigor is not merely a technical consideration but a determinant of credibility. Regulators, journal reviewers, and clinicians increasingly scrutinize whether studies are appropriately powered for their stated objectives and whether conclusions are supported by pre-specified analyses. Trials that demonstrate hypothesis discipline and transparent statistical planning are more likely to generate evidence that is reproducible, interpretable, and suitable for substantiating therapeutic or preventive claims.

**Figure 2:**
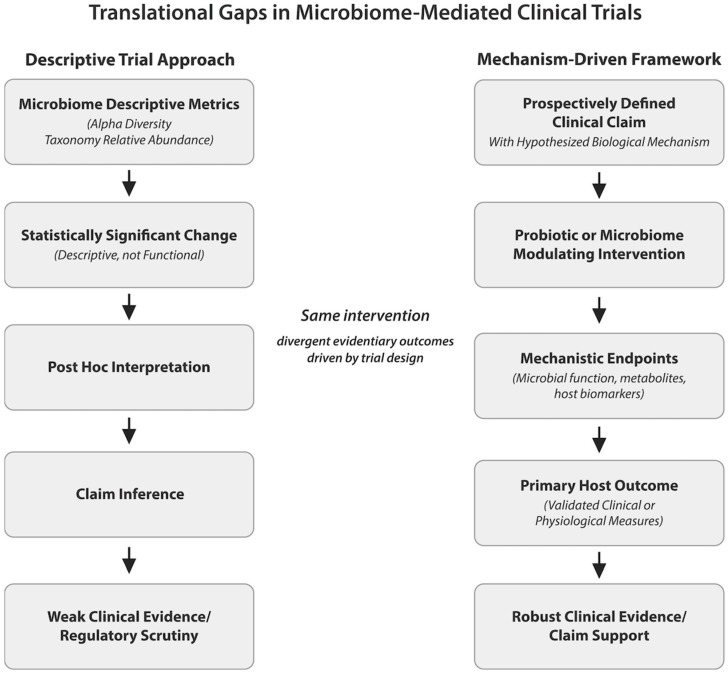
Translational gaps in microbiome-mediated clinical trials arising from descriptive versus mechanism-driven study designs. The left panel illustrates a common descriptive approach in which changes in microbiome composition or diversity are treated as surrogate indicators of efficacy, often leading to post hoc interpretation and limited clinical or regulatory support. The right panel depicts a mechanism-driven framework in which a prospectively defined clinical claim guides endpoint selection, integrating microbiome measures as mechanistic mediators alongside host biomarkers and validated clinical outcomes. Although the intervention may be identical, differences in study design and endpoint hierarchy substantially influence interpretability, translational relevance, and claim robustness.

In the context of probiotic and microbiome-targeted interventions, acknowledging and accommodating microbiome variability through disciplined hypothesis selection, realistic power calculations, and staged development approaches can substantially improve the reliability of clinical validation efforts. Such practices help ensure that observed effects reflect true biological signals rather than statistical artifacts and that conclusions drawn from complex datasets are proportionate to the strength of the evidence. These contrasting design logics—and their implications for interpretability and clinical validation—are summarized schematically in Figure 2.

## 9. Reframing the Microbiome as a Mechanistic Mediator

A recurring source of confusion in probiotic and microbiome-focused clinical research is the treatment of the microbiome itself as a primary outcome of interest. While changes in microbial composition or diversity are often highlighted as indicators of efficacy, health claims are ultimately made about host benefit rather than microbial state. This disconnect contributes to the overinterpretation of microbiome data and weakens the translational relevance of many trials.

In most clinical contexts, the gut microbiome functions not as an endpoint, but as a mechanistic intermediary between an intervention and a host outcome. Probiotic or nutraceutical interventions may alter microbial composition or function, which in turn influences host physiology through effects on metabolism, immune signaling, barrier integrity, or inflammatory tone. The clinical relevance of microbiome modulation therefore depends on whether these downstream host effects are demonstrated, not merely whether microbial change occurs.

When microbiome measures are treated as endpoints rather than mechanisms, trials risk conflating change with benefit. For example, a statistically significant shift in microbial taxa may be biologically interesting, yet clinically inconsequential if it does not lead to measurable improvement in relevant host outcomes. Conversely, meaningful host benefits may occur with minimal or transient changes in microbial composition, particularly when functional activity rather than taxonomic structure is the primary driver of effect [68,69,70].

Viewing the microbiome as a mechanistic mediator has important implications for trial design and interpretation. First, it clarifies the role of microbiome data within the evidentiary hierarchy. Microbiome analyses are most powerful when used to explain how an intervention exerts its effects, rather than to define whether it is effective. Second, this framing encourages integration of microbial data with host-level biomarkers and clinical endpoints, strengthening biological plausibility and causal inference.

This perspective also helps reconcile inconsistencies across studies. Variability in microbiome composition, sequencing methods, or analytical pipelines may lead to divergent descriptive findings, even when interventions produce similar host-level effects. By prioritizing host outcomes and mechanistic integration over descriptive microbiome change, researchers can better assess the clinical significance of probiotic interventions across diverse populations and study designs [71,72].

Importantly, reframing the microbiome as a mediator does not diminish its scientific importance. On the contrary, it elevates microbiome research by situating microbial data within a coherent biological narrative that links intervention, mechanism, and outcome. Trials designed with this perspective are more likely to generate interpretable, reproducible evidence and to support claims that are credible to clinicians, regulators, and patients.

As the field moves toward more clinically oriented applications of probiotics, adoption of this mechanistic framing will be increasingly important. Recognizing the microbiome as a mediator rather than an endpoint provides a conceptual foundation for more disciplined trial design and sets the stage for developing robust frameworks for clinical validation.

## 10. Toward More Robust Frameworks for Clinical Validation of Probiotics

**Figure 3:**
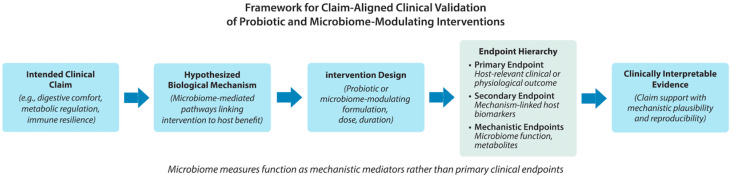
Conceptual framework for claim-aligned clinical validation of probiotic and microbiome-modulating interventions. The framework emphasizes prospective definition of the intended clinical claim, articulation of hypothesized microbiome-mediated mechanisms, disciplined intervention design, and hierarchical endpoint selection prioritizing host-relevant outcomes. Microbiome measures are positioned as mechanistic mediators supporting biological plausibility rather than as primary indicators of efficacy.

The recurring limitations discussed above point to a central conclusion: many probiotic and microbiome-focused clinical trials fail to support defensible claims not because interventions lack biological activity, but because study designs do not adequately align clinical claims, mechanistic hypotheses, endpoints, and analytic interpretation. Addressing this gap requires a more disciplined and translational approach—one that treats trial design as an exercise in mechanistic validation rather than exploratory data collection. A structured framework for claim-aligned validation of probiotic and microbiome-modulating interventions is illustrated in Figure 3.

A useful starting point is explicit definition of the clinical claim. Whether an intervention is proposed to support digestive function, metabolic health, immune resilience, or disease risk reduction, the claim should be prospectively defined in terms of a measurable host benefit. This anchors all downstream design decisions and helps prevent retrospective outcome interpretation. Vague or overly broad claims tend to result in diffuse endpoint selection and ambiguous conclusions.

Once the intended claim is articulated, investigators should identify the biological mechanisms through which the intervention is hypothesized to act. In microbiome-focused trials, this often involves specifying how microbial modulation (e.g., altered fermentation, immune signaling, barrier function) leads to physiological change. This mechanistic rationale guides endpoint selection and informs analytical strategy.

Endpoints should then be organized hierarchically. Primary endpoints must directly assess the host-level benefit aligned with the claim. Secondary endpoints may include host biomarkers reflecting mechanistic engagement. Microbiome metrics—whether taxonomic, functional, or metabolic—should be positioned as mechanistic mediators, used to interpret how the intervention may be working, rather than as outcomes that define efficacy.

To operationalize this framework, a range of analytical tools can support mechanistic inference:

• Mediation analysis (e.g., structural equation modeling, counterfactual frameworks, or causal mediation via the Baron and Kenny method) can statistically test whether microbiome changes mediate observed host effects.
• Longitudinal sampling and multi-omics integration (e.g., combining metagenomics, metabolomics, and transcriptomics) can strengthen temporal and functional linkage.
• Functional microbiome measures—such as SCFA production, bile acid metabolism, or immune-modulatory gene expression—offer more direct evidence of mechanistic engagement than taxonomic shifts alone.
• Supportive criteria for mechanistic inference include dose–response relationships, temporal precedence, and strain-specific biological activity replicated in independent studies.

Equally important is statistical and methodological discipline. Sample size calculations must be based on expected effect sizes for primary outcomes. Pre-specification of secondary and exploratory analyses prevents overinterpretation. Pilot studies can inform endpoint selection and variability prior to confirmatory trials.

Attention to trial execution and confounder control is also critical. Dietary patterns, medications, comorbidities, and lifestyle behaviors should be explicitly managed at the design stage, not treated as nuisance variables in post hoc analyses. Standardized sample handling and transparent protocol reporting enhance reproducibility and credibility.

Finally, interpretation must remain proportionate to evidentiary strength. Demonstrating microbiome modulation alone is insufficient for clinical claims. Conversely, when host improvements occur in parallel with mechanistically consistent microbiome changes, confidence in causal inference is strengthened. Clear distinctions between exploratory and claim-supportive evidence protect scientific integrity and improve translatability.

Together, these principles define a framework for more robust clinical validation of probiotics and microbiome-modulating interventions. By aligning claims with mechanisms, prioritizing meaningful endpoints, and integrating microbiome data mechanistically rather than descriptively, future trials can generate evidence that is clinically relevant, reproducible, and credible.

## 11. Conclusions and Future Directions

The growing clinical and commercial interest in probiotics and other microbiome-modulating interventions reflects genuine promise for therapeutic and preventive applications across diverse conditions. However, the persistent gap between reported microbiome changes and defensible health claims indicates that the central barrier to validation is often methodological rather than biological. As argued in this Commentary, many trials are undermined by modifiable design and interpretive choices that reduce clinical relevance, weaken causal inference, and limit reproducibility.

Across the current literature, recurring pitfalls include overreliance on descriptive microbiome metrics as surrogate indicators of benefit, misalignment between prespecified endpoints and the claims ultimately advanced, and excessive dependence on symptom-only outcomes in settings characterized by substantial placebo responsiveness. These challenges are compounded by inadequate control of confounding variables—particularly diet, antibiotic exposure, and concomitant medications—as well as by endpoint overload, underpowered designs, and insufficient statistical discipline. Collectively, these issues can produce datasets that are statistically interesting yet clinically ambiguous, limiting their value for rigorous validation.

A unifying corrective principle is to treat the microbiome primarily as a mechanistic mediator rather than the endpoint of interest. Clinical claims should be anchored in host-relevant outcomes and supported by prospectively defined endpoints aligned with explicit biological mechanisms. Microbiome measures are most informative when used to explain how an intervention engages its proposed mechanism and when interpreted alongside objective host biomarkers that anchor symptom changes in physiology.

Future progress will depend on adopting more disciplined validation pathways. This perspective is consistent with recent patent-landscape analyses that highlight rapid advances in diagnostic technologies, multi-omics integration, precision microbial therapeutics, synthetic biology tools, and personalized microbiome-based interventions, underscoring the need for rigorous clinical validation frameworks that can keep pace with innovation (D’Urso et al., [73]). In parallel, advances in artificial intelligence are transforming microbiome research by enabling the integration and interpretation of complex multi-omics datasets. AI-based approaches—including machine-learning models applied to metabolomic, transcriptomic, proteomic, and metagenomic data—are now capable of identifying functional interaction networks, predicting microbiome–host associations, and supporting the development of targeted microbial therapies. Recent analyses highlight the increasing use of AI tools in guiding probiotic and synbiotic interventions, improving classification of dysbiosis states, and informing precision-based treatments across a range of clinical conditions [74].

Early-stage studies should be used to refine mechanistic hypotheses, identify appropriate biomarkers, and estimate variability, thereby informing adequately powered confirmatory trials. Trial designs should incorporate transparent strategies for managing confounders, emphasizing baseline characterization, protocol adherence, and standardized sampling workflows. Equally important, interpretation should remain proportional to evidentiary strength, clearly distinguishing exploratory observations from claim-supportive outcomes.

As the field matures, greater rigor should not be viewed as a constraint on innovation but as a prerequisite for meaningful translation. By aligning claims, mechanisms, and endpoint hierarchies—and by integrating microbiome measures with host biomarkers and clinically meaningful outcomes—microbiome-mediated trials can move beyond descriptive change toward reproducible clinical validation. This shift is essential for realizing the therapeutic and preventive potential of probiotics and related interventions and for ensuring that clinical applications are supported by evidence that is credible to clinicians, regulators, and scientific peers.

## Figures and Tables

**Table 1:** Selected Clinical Trials Illustrating Microbiome-Focused Pitfalls.

Citation	Intervention Tested	Microbiome Endpoints	Reported Health Claim	Key Methodological Issue
Meiners et al., 2025 [28]	Fiber and polyphenol-rich diets	Fatty-acid producing bacteria	Increased visceral fat loss mediated by urolithins, and enhanced anti-inflammatory effects,	Descriptive endpoints as surrogates
Zhang et al., 2019 [29]	Navy bean (fiber-rich) diet vs. habitual diet (8 weeks) in obese colorectal cancer survivors	Gut microbiome α-diversity; beneficial genera (e.g., *Faecalibacterium*, *Bifidobacterium*); opportunistic bacteria.	Improved gut health and potential cancer prevention–diet-induced microbiome shifts associated with reduced inflammation and cancer risk markers.	Descriptive endpoints as surrogates
Wastyk et al., Cell, 2021 [30]	Diet high in fermented foods vs. high-fiber diet (10 weeks) in healthy adults	Microbial diversity (Shannon index) in fermented-food group; no change in diversity with high-fiber diet; compositional shifts and altered microbial functional genes.	Reduced inflammation and improved immune responses–fermented-food diet lowered 19 inflammatory blood markers (e.g., IL-6) and modulated immune cell activation.	Reliance on broad metrics:
Wastyk et al., Gut Microbes, 2023 [31]	Multistrain probiotic vs. placebo (18 weeks) in adults with metabolic syndrome	Longitudinal stool profiling: no significant overall change in microbiome composition or diversity across the full cohort; post hoc “responder” subgroup had distinct microbial shifts after probiotic (vs. non-responders).	Metabolic improvements (subset only): no overall effect on insulin resistance or lipids, but responders showed triglycerides and blood pressure. Diet was a differentiating factor, suggesting probiotic efficacy tied to microbiome-diet interactions.	Endpoint misalignment and post hoc narrative
El-Salhy et al., Gut, 2020 [32]	Fecal microbiota transplant (FMT) from one healthy donor vs. placebo (own stool) in IBS (single delivery + capsules)	Microbial richness and diversity in FMT groups; major shifts toward donor-like taxonomic profile in FMT recipients (vs. minimal change with autologous placebo).	Symptom relief in IBS–8-week responder rates: 77% (FMT low dose) and 89% (FMT high dose) vs. 24% placebo, with improved IBS symptom scores and quality of life.	Functional ambiguity despite microbiome change.
Dewulf et al., Gut, 2013 [33]	Prebiotic inulin-type fructans vs. placebo (3 months) in obese women	Abundance of putative beneficial taxa (*Bifidobacterium*, *Faecalibacterium prausnitzii*); relative abundance of *Bacteroides* spp.; no global community shift by PCA.	Improved metabolic health markers–slight reduction in body fat and metabolic endotoxemia (serum LPS), with correlations noted between microbial changes and lowered plasma inflammation/metabolite levels.	Modest outcomes with correlative
Ghosh et al., Gut, 2020 [34]	Mediterranean diet vs. regular diet (1 year) in elderly adults (five-country NU-AGE study)	Fiber-degrading SCFA-producing genera with diet adherence; overall β-diversity shift in gut microbiome; enriched taxa (e.g., *Faecalibacterium*, *Roseburia*) correlated with diet compliance.	Reduced frailty and improved cognitive function–better diet adherence associated with lower frailty index and higher cognitive scores, alongside reduced inflammatory markers (CRP, IL-6).	Associative claim, mechanism not established.
Staudacher et al., Gastroenterology, 2017 [35]	Low-FODMAP diet vs. sham diet (4 weeks) in IBS patients; plus a probiotic or placebo sub-arm	Co-primary endpoints included stool *Bifidobacterium* abundance (qPCR): low-FODMAP diet caused *Bifidobacterium* counts (vs. baseline and vs. sham) and did not change diversity. Adjunct probiotic restored *Bifidobacterium* levels.	Improved IBS symptoms–61% on low-FODMAP diet reported adequate symptom relief vs. 39% on sham diet (per-protocol). Probiotic co-administration aimed to mitigate microbiome “downsides” of the diet (i.e., counteract loss of bifidobacteria) without hindering symptom benefits.	Endpoint trade-off and misalignment.
John et al., Front. Microbiomes, 2024 [24]	Multi-strain probiotic vs. placebo during antibiotic [36] therapy (single-center RCT)	Antibiotic disruption of gut flora: probiotic group preserved overall microbiota α-diversity (no post-antibiotic diversity loss, vs. significant diversity drop in placebo). Probiotic maintained higher Bacteroides and lower Enterobacteriaceae, and attenuated expansion of antibiotic resistance genes (ARGs).	Mitigation of antibiotic-induced dysbiosis–probiotic ostensibly protected “gut health” by maintaining microbial balance and reducing the antibiotic resistance gene reservoir. Implied benefit: lower risk of antibiotic-associated complications (e.g., diarrhea or resistance development).	Surrogate endpoints & implied benefit.
Depommier et al., Nat. Medicine, 2019 [36]	Pasteurized *Akkermansia muciniphila* supplement vs. placebo (3 months) in overweight/obese insulin-resistant adults	No significant change in overall gut microbiome composition or diversity (the introduced bacterium did not colonize or alter community structure). [*A. muciniphila* detectable but low in stool of treated subjects.] Secondary endpoint: gut barrier integrity (plasma LPS).	Improved metabolic markers–pasteurized *Akkermansia* led to better insulin sensitivity (+~29%), lower insulinemia (−34%) and reduced blood cholesterol, without weight loss. Additionally, modest reductions in inflammation and liver enzymes were noted, suggesting metabolic health benefits despite no microbiota upheaval.	Lack of mechanistic.
Costello et al., JAMA, 2019 [37]	Donor fecal microbiota transplant vs. autologous placebo (1-week intensive protocol) in mild-moderate ulcerative colitis	Engraftment of donor microbial strains and community diversity in FMT group (per protocol analysis in study manuscript); microbiome shift toward “healthier” profile in responders (reported in secondary analyses).	Induction of remission in ulcerative colitis–at 8 weeks, 32% achieved steroid-free clinical remission with donor FMT vs. 9% with placebo (autologous FMT). Some responders maintained remission at 12 months.	Outcome achieved, mechanism unclear.

Abbreviations: RCT—randomized controlled trial; IBS—irritable bowel syndrome; FMT—fecal microbiota transplantation; SCFA—short-chain fatty acid; LPS—lipopolysaccharide (endotoxin); IL-6—interleukin 6; CRP—C-reactive protein.

## Data Availability

Not applicable.

## Abbreviations

The following abbreviations are used in this manuscript:

PRO	Patient-Reported Outcomes
SCFA	Short-Chain Fatty Acids
IRB	Institutional Review Board
CRediT	Contributor Roles Taxonomy
